# The Effect of Microwave Oven Drying on the Compressive Strength of Type III and IV Dental Stones at Different Time Intervals

**DOI:** 10.2174/1874210601812010494

**Published:** 2018-07-31

**Authors:** Suha Fadhil Dulaimi, Sabiha Mehdi Kanaan

**Affiliations:** Department of Prosthodontics Technologies, College of Health and Medical Technologies, Middle Technical University, Ministry of Higher Education and Scientific Research, Baghdad, Iraq

**Keywords:** Microwave irradiation, Dental stone, Compressive strength, Cross-contamination, Prosthodontics practice, Polystyrene split mold

## Abstract

**Background:**

Dental stone cast needs 24 to 48 hours to evaporate excess water and acquire enough strength for manipulation. The microwave oven is used to save time by drying and disinfecting stone casts.

**Objectives:**

The aim of the present study is to evaluate the effect of microwave oven drying on the compressive strength of Type III and Type IV dental stone in three time intervals-3 hours, 5 hours and 24 hours after pouring.

**Methods:**

The study involved 120 samples: 60 samples for each type of dental stone. Thirty samples were subjected to air drying at 3, 5 and 24 hours) and 30 samples were dried in the microwave oven at 2450 MHZ -900 W for 150 seconds (at 3, 5 and 24 hours). The samples were tested by unconfined compression machine, with a 2000 kg proving ring at cross-head speed of 1 mm/min.

**Results:**

For the Type III dental stone drying at 3 hours, microwave radiation causes a significant increase in compressive strength and shows the highest score (mean 125 kg/cm); however, at 5 hours no significant difference was observed. However, at the 24 hour time interval, microwave drying causes significant reduction in the mean (100 kg/cm) *p*<0.01 .For the Type IV dental stone testing at all-time intervals (3,5, 24 hours), a significant reduction (*p*<0.05) in mean values was shown(80,85,87 respectively).

**Conclusion:**

Microwave oven drying increased the compressive strength of Type III dental stone at the 3 hour time interval from pouring, while microwave drying at the 5 hour interval had no significant effect; and at 24 hour the compressive strength of the dental stone was reduced. For the Type IV dental stone microwave drying had a detrimental effect on compressive strength at all-time intervals.

## 1 INTRODUCTION

Cross-contamination in prosthodontics practice can be prevented by microwave irradiation of stone cast [1]. At the same time, the stone cast should exhibit enough compressive strength and abrasion resistance to withstand the carving process, particularly at the margin [2]. Freshly poured stone cast needs 24-48 hours to lose excess water and gains enough surface hardness and compressive strength to be manipulated without damage [3]. 

However, in clinical and laboratory practice, it is sometimes necessary to save drying time and handle the stone cast sooner after its fabrication. In addition, there is a need to disinfect the stone cast. Previous studies used microwave irradiation to reduce the drying time with different microwave drying protocols [4, 5].

In 1985, Luebke and Scheinder [6] were the first to investigate the effect of microwave oven drying on gypsum products. They concluded that refractory casts could be dried in microwaves which would shorten the fabrication time but that extremely wet or water-soaked casts must not be dried by microwave oven because of rapid boiling of excess water causing cracks in the casts. Other researchers found that high power microwave irradiation had a detrimental effect on the compressive strength of Type IV dental stone and no significant effect on Type III dental stone 2-4 hours after pouring [4].

Meanwhile, Heresek *et al*. [5] found variations in compressive strength of the available commercial versions of Type IV dental stone. The recent research stated that microwave irradiation after one hour from mixing of Types III and IV significantly reduces compressive strength and surface detail [7].

To the best of the authors' knowledge, to date, the appropriate time for microwave drying of a stone cast after pouring without harmful effect on compressive strength and the type of dental stone used have not been investigated.

The aim of the present study is to investigate the effect of microwave drying on compressive strength of Type III and IV dental stone at 3, 5 and 24 hour time intervals.

## 2 MATERIALS AND METHODS:

### 2.1 Preparation of Test Samples

**Fig. (1):**
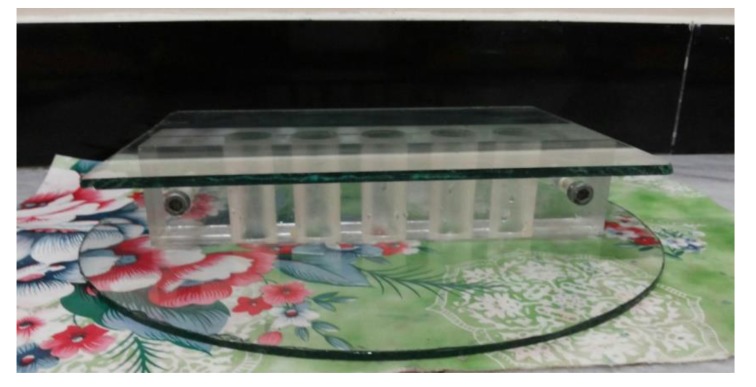
A special split mold used to prepare (5) cylindrical specimens.

For compressive strength, a polystyrene split mold (Fig. **1**) was manufactured according to ADA specification No.25 for dental gypsum products [6]. The mold prepared five samples, each measuring 20 mm in diameter and 40 mm in length.

Dental stone was mixed according to the manufacturer's water/powder ratio. To reduce porosity, rubber bowel was placed on the vibrator for 30 seconds to remove air bubbles from the stone mix [8]. The assembled polystyrene mold positioned on glass slab and stone slurry poured into the mold.

### 2.2 Samples Grouping

There were a total of 120 samples, 60 samples of Type III and 60 of Type IV. Thirty were tested for each dryness method. For the air-dried groups, the samples were left to dry in air at 20 ± 2 ºC for 3, 5 and 24 hours after pouring. For the microwave group, the samples were dried in the microwave oven at 2450 MHZ, 900 W for 150 seconds; the samples were then reversed to irradiate the other part for another 150 seconds [1].

### 2.3 Testing Procedure

**Fig. (2):**
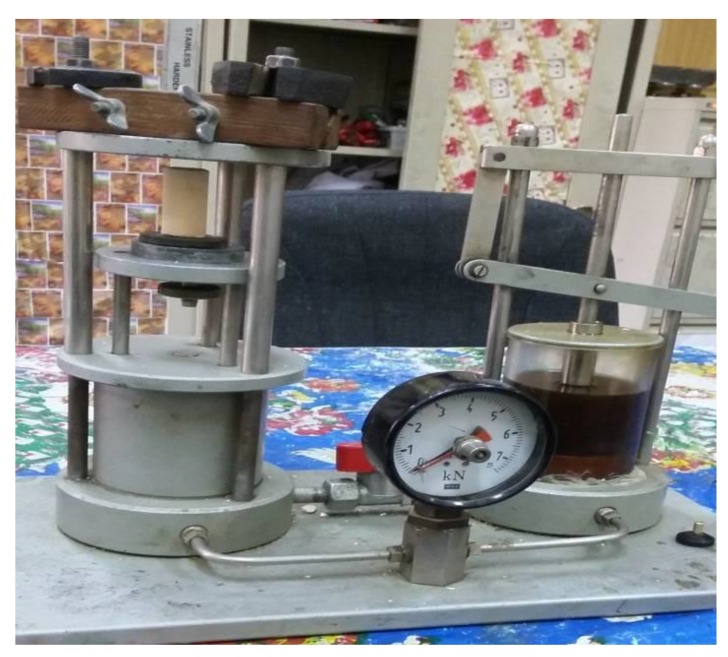
Unconfined compressive machine.

The samples were tested by unconfined compression machine (Inc, model CN 472, EVANSTON III USA) with a 2000 kg proving ring at a cross-head speed of 1 mm/mint (Fig. **2**).

## 3 RESULTS

**Fig. (3):**
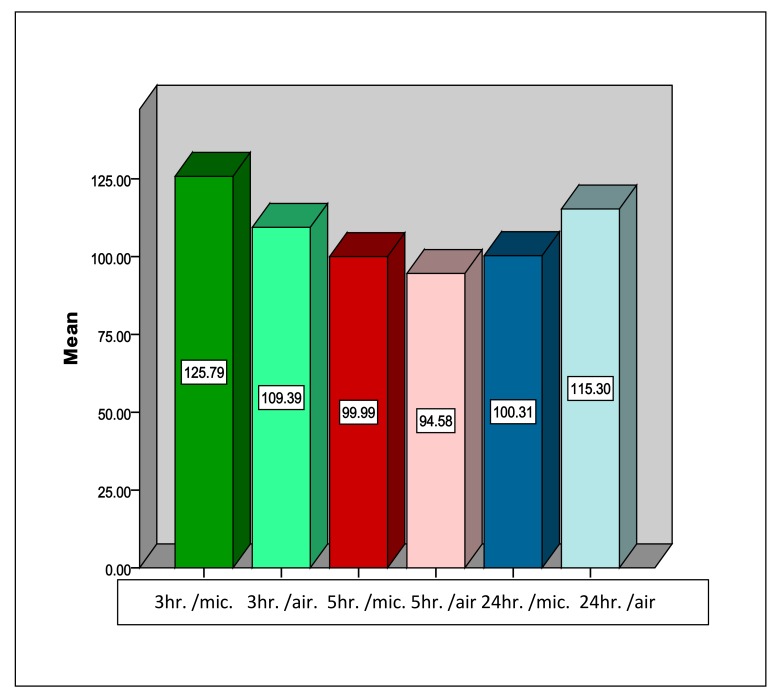
Bar chart of mean compressive strength of Type III dental stone-dried in microwave oven (Mic.) and groups air-dried (Air) at three time intervals from pouring.

Descriptive statistics of the results of this study for the Type III dental stone are shown in Table **1**. The highest mean value was found in the group of stone samples dried in the microwave oven at 3-hour-time intervals. Inferential statistics using the student t-test showed that for the Type III dental stone at 3 hours, microwave irradiation caused a significant increase (mean 125 g/cm^2^) in the compressive strength, while at 5 hours drying, no significant difference was recorded. However at the 24 hour time interval, microwave drying (100 kg/cm^2^) caused a significant reduction in compressive strength in comparison to air drying with a *P*-value < 0.01 (Table **2** and Fig. **3**).

For Type IV dental stone, the descriptive statistics in Table **3** and Fig. (**4**) show a reduction in the mean of compressive strength for all time intervals tested. Student t-test showed that microwave drying at all-time intervals (3, 5, 24 hour) caused a significant reduction in compressive strength in comparison to air drying, as shown in Tables **3**, **4** and Fig. (**4**).

## 4 DISCUSSION

Dental stone is routinely used in dental practice for fabrication of indirect restoration. Dental stone whether Type III or Type IV should exhibit high strength, superior abrasion resistance and minimal setting expansion. Literatures and stone manufacturers recommend waiting for 24-48 hours before manipulating with the cast to permit evaporation of excess water necessary for adequate mechanical properties [9].

Previous research studied different kinds of gypsum products that were dried in the microwave oven. Such studies found that new, wet and water soaked casts should not be dried in a microwave oven because the rapid boiling of water particles causes cracks in the casts [6].

In our study, microwave irradiation protocol was investigated by Goel *et al*. [1] who placed stone casts samples in a household microwave oven and irradiated them at 2450 MHZ 900W for 150 seconds. The samples were then reversed and the other part was irradiated for another 150 seconds. The research concluded that the previous irradiation protocol is considered better disinfection method in comparison with chemical disinfection.

For the Type III dental stone, our study shows that microwave irradiation at 3 hours causes a significant increase in compressive strength mean (125 g/cm^2^) due to the removal of excess water and does not affect the hemihydrate crystals conversion into dehydrate. At the 5 hour-interval, no significant difference was found in microwave irradiation compared to air drying. However at 24 hours, microwave drying caused a significant reduction in compressive strength . High-power microwaving was found to reduce compressive strength of Type III and Type IV by Tuncer *et al.* [4]. For the 24 hour interval, Luebke and Schneider [6] found no significant difference between air drying and microwave drying for the Type III stone with high-power microwaving. Malaviya *et al.* [7] concluded that microwave irradiation after one hour from pouring reduced the strength of both types of dental stones significantly because the casts were extremely wet; this caused rapid boiling of the free water, which meant that the conversion of hemihydrates to dihydrate crystals was not completed [6, 8].

Hasan and Mohamed [8] agree with present study that, at 24 hour, microwaving of Type III stone reduces the compressive strength of the material; this may be due to surface porosity and escape of free water from the stone surface during exposure to microwave irradiation that would generate cracks and holes creating surface porosity [10]. For the Type IV dental stone, microwaving caused a significant reduction at all time intervals.

These findings are in agreement with Malaviya *et al*.'s results at 1 hour interval but disagree with their findings at the 24 hour interval [7]. They are also in conflict with da Silva *et al*. [11] who found that microwave irradiation does not affect the compressive strength of Type IV dental stone at 2 hour intervals.

The reasons for these conflicting results in our study and previously mentioned studies could be explained by the environmental conditions during testing and the storage environment which are factors that might affect the strength of stone [12]. In addition, the microwave protocol used in this study [1] differs from other studies.

## CONCLUSION

With the limitation of the present study, the following conclusions can be drawn:

Microwave oven drying of Type III dental stone increased its compressive strength at 3 hours after pouring, had no effect at the 5 hour interval and caused a reduction in compressive strength at the 24 hour interval. Therefore, microwave oven drying is not recommended for Type IV dental stone because it decreased the compressive strength of the stone at all-time intervals tested.

## Figures and Tables

**Fig. (4):**
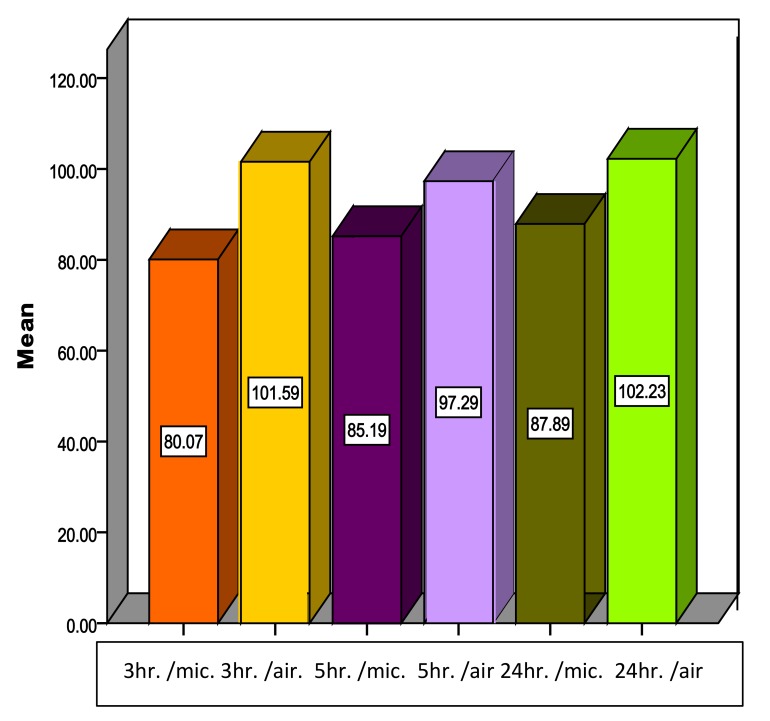
Bar chart of mean compressive strength of Type IV dental stone dried with microwave oven (Mic.) and groups dried with air (Air) at three-time intervals from pouring.

**Table 1:** Descriptive statistics of compressive strength of Type III dental stone groups dried with microwave oven (Mic.) and groups dried with air (Air) at three time intervals from pouring.

**Groups**	**N**	**Min.**	**Max.**	**Mean**	**Std. Deviation**
Type III/ Time3 hr/ Mic.	10	106.68	141.71	125.7900	13.88118
Type III/Time3 hr / Air.	10	98.72	121.01	109.3900	7.98052
Type III /Time5 hr /Mic.	10	71.65	114.64	99.9940	12.93140
Type III /Time5 hr /Air.	10	85.98	101.91	94.5810	5.42448
Type III /Time24 hr /Mic.	10	90.76	108.28	100.3140	5.95844
Type III /Time24 hr/ Air.	10	108.28	121.01	115.2990	4.55980

**Table 2:** t-test between groups (Type III dental stone).

**Groups**	**t**	***P*- Value**	**C.S**
Type III/ Time3 hr./Mic. - Type III /Time3 hr. /Air.	3.611	.006	*P*<0.01 (HS)
Type III/ Time5 hr./ Mic. - Type III /Time5 hr. /Air.	1.506	.166	*P*>0.05(NS)
Type III/ Time24 hr./ Mic. - Type III/Time24 hr. /Air.	6.147	.000	*P*<0.01 (HS)

**Table 3:** Descriptive Statistics of compressive strength of Type IV dental stone groups dried in a microwave oven (Mic.) and groups dried with air (Air) at three-time intervals from pouring.

Groups	N	Min.	Max.	Mean	Std. Deviation
Type IV/ Time3 hr./ Mic.	10	70.06	95.54	80.0680	7.49580
Type IV/Time3 hr. / Air.	10	82.80	114.64	101.5870	9.98026
Type IV/ Time5 hr. /Mic.	10	79.61	93.94	85.1850	4.99197
Type IV / Time5 hr. /Air.	10	92.35	101.91	97.2890	3.86261
Type IV / Time24 hr./ Mic.	10	79.61	114.64	87.8910	10.60944
Type IV/ Time24 hr. / Air.	10	76.43	121.01	102.2250	14.96978

**Table 4:** t-test between groups (Type-IV).

**Groups**	**t**	***P*- Value**	**C.S**
Type IV/ Time3 hr./ Mic.- Type IV/ Time3 hr./Air.	7.478	0.000	*P*<0.01 (HS)
Type IV/ Time5 hr./ Mic. - Type IV / Time5 hr. / Air.	5.019	0.001	*P*<0.01 (HS)
Type IV / Time24 hr./ Mic.- Type IV / Time 24hr./ Air	2.701	0.024	*P*<0.05(S)
